# Stereological sampling-based estimates of dopaminergic and cholinergic neuron numbers in young adult and aged male and female Fischer 344 × Brown Norway F1 hybrid rats

**DOI:** 10.3389/fnagi.2026.1730515

**Published:** 2026-08-03

**Authors:** Hannah E. Phelps, Merritt R. Reese, Swetha Jeyagopal, Aryan Sahota, Wonn S. Pyon, Jennifer L. Bizon, Barry Setlow, Matthew R. Burns

**Affiliations:** 1 University of North Carolina School of Medicine, Chapel Hill, NC, United States; 2 Department of Neurology, University of Florida College of Medicine, Gainesville, FL, United States; 3 Department of Neuroscience, University of Florida College of Medicine, Gainesville, FL, United States; 4 McKnight Brain Institute, University of Florida, Gainesville, FL, United States; 5 Center for Cognitive Aging and Memory, University of Florida, Gainesville, FL, United States; 6 Department of Psychiatry, University of Florida College of Medicine, Gainesville, FL, United States; 7 Department of Neurology, Norman Fixel Institute for Neurological Diseases, University of Florida College of Medicine, Gainesville, FL, United States

**Keywords:** aging, cholinergic, dopaminergic, rat, sex differences, stereological sampling

## Abstract

Age is the primary risk factor for neurodegenerative disorders such as Parkinson’s disease and Alzheimer’s disease, which are characterized by profound loss of midbrain dopaminergic neurons and basal forebrain cholinergic neurons, respectively. In addition, loss of these neurons can occur even in “normal” (i.e., non-pathological) aging, and as a result there is growing recognition of the value of aged animals in studying both normal aging and age-related neurodegenerative disease. The Fischer 344 x Brown Norway F1 hybrid (FBN) rat is commonly used for such purposes and is recommended for use in aging studies by the NIH due to its extended lifespan and more accurate representation of age-related human diseases such as sarcopenia and cognitive decline. Despite the significant value and broad use of this model, dopaminergic and cholinergic neurons have not been well characterized in this strain, and there have been even fewer comparisons between males and females. To address this issue, we used systematic random sampling principles to generate population estimates for dopaminergic neurons in the substantia nigra pars compacta and ventral tegmental area, and cholinergic neurons in the medial septum and vertical limb of the diagonal band of Broca in 5–6.5-month and 20–26-month-old FBN rats of both sexes. Dopaminergic and cholinergic cell bodies were immunolabeled for tyrosine hydroxylase and choline acetyltransferase, respectively. Because the final counts were obtained from full-thickness image stacks using cell-profile inclusion rules and relatively sparse section sampling, these values are presented as stereological sampling-based estimates rather than strict design-based stereological totals. Within these methodological limits, no age differences in dopaminergic or cholinergic neuron-number estimates were detected in any region assessed, nor were there differences between males and females. These data suggest that FBN rats can be used to model induced age-related neurodegenerative disease without the potential confound of a large independent age-associated loss of these dopaminergic or cholinergic neuronal populations.

## Introduction

Investigation of aging and its impacts on the central nervous system has become increasingly relevant as an aging population and the risk for neurodegenerative disease grow side by side. Aging is associated with brain atrophy (Erten-Lyons et al., 2013; Smith et al., 2004), increases in number of plaques and tangles characteristic of Alzheimer’s disease (Avila et al., 2004; Guillozet et al., 2003), and age-related cognitive decline (Lee and Kim, 2022; Schaie, 1996; Gazzaley et al., 2005; Flicker et al., 1984). Cognitive decline that occurs with age in humans is associated with changes in dopaminergic and cholinergic neuron function and number, and these changes are also closely linked to a variety of age-related diseases (e.g., Karrer et al., 2017; Ma et al., 1999; Mitsis et al., 2009; Grothe et al., 2013).

Dopamine signaling dysfunction is associated with Lewy Body diseases such as Parkinson’s disease (PD) and Dementia with Lewy Body, attention deficit hyperactivity disorder (ADHD), schizophrenia, depression, and substance use disorders (Klein et al., 2019), and the prevalence of many of these conditions changes with age. In the normal human aging process, it is well characterized that global brain dopamine activity decreases with age, with both synaptic functional changes and cell loss implicated (Karrer et al., 2017; Ma et al., 1999; Buchman et al., 2012). Changes in the cholinergic system including cholinergic cell loss have been described with PD and Alzheimer’s disease (AD), as well as non-pathological aging (Perry, 1980; Bigl et al., 1987; Mitsis et al., 2009; Grothe et al., 2013; Lagarde et al., 2017). Acetylcholine is responsible for a variety of functions, including motor control, memory, and cognition (Sam and Bordoni, 2024). Rats are useful models to study aging and neurodegenerative disease, due to their relatively large body and brain size (allowing for feasible anatomic targeting for stereotactic injection and optogenetic and fiber photometry leads, for example) and capacity to perform sophisticated motor and cognitive tasks. As such, it is important to understand how dopaminergic and cholinergic function changes with age in this species. Notably, the degree of nigral and basal forebrain neuron loss in aging humans varies substantially between individuals, with some older adults showing relatively preserved cell numbers despite measurable functional decline (e.g., Buchman et al., 2012; Grothe et al., 2013).

Within the literature investigating the impact of age on dopaminergic and cholinergic neurons in rat models, there is disagreement on the type and extent of age-related differences. A study in young (5–6 months) and aged (24–25 months) male Sprague Dawley rats found no changes with age in number of dopaminergic neurons, neuronal area, or neuronal length in midbrain regions A8, A9, or A10 (Emerich et al., 1993). In a later study using female Sprague Dawley rats of three age groups - young (5–6 months), old (22–24 months), and very old (30–32 months) - there were no differences in ventral tegmental area (VTA) dopamine neuron counts but there was a significant decrease in substantia nigra pars compacta (SNc) dopamine neurons in the very old compared to young and old rats (Sanchez et al., 2008). Additional studies have also come to conflicting conclusions regarding age changes in dopamine neuron number (Mizoguchi et al., 2009; Pereira et al., 2021), rendering it important to reevaluate this issue, particularly in the context of changes in laboratory rodent health and care over the past several decades. Taken together, these reports range from little or no detectable age-related loss of midbrain dopamine neurons to modest, regionally restricted decreases that emerge only at very advanced ages, underscoring the extent to which strain, age range, and methodological approach can shape the apparent impact of aging on this system.

There is a similar lack of consensus in the literature with regard to age changes in cholinergic neurons in rats. The first study to evaluate cholinergic cell numbers in aged rats (Fischer et al., 1992) found a significant reduction in basal forebrain cholinergic cell numbers in 24-month and 30-month old compared to 3-month old female Sprague–Dawley rats. Similarly, De Lacalle and colleagues found that choline acetyltransferase (ChAT) immunoreactive neuron numbers were significantly decreased in aged (24-33-month old) compared to young (4-6-month old) male Sprague–Dawley rats across basal forebrain nuclei (de Lacalle et al., 1996). Similar results were found in male Wistar rats (Niewiadomska et al., 2000), male Long-Evans rats (Baskerville et al., 2006) and male Fischer 344 rats (Bañuelos et al., 2013). In contrast to these findings in basal forebrain, a more recent study found that the numbers of ChAT positive neurons in the pedunculopontine and laterodorsal tegmental nuclei were not significantly different between young and aged male Wistar rats (Pereira et al., 2021), although there were some age changes in neuronal morphology.

The current study aimed to address several needs in the existing literature regarding the effects of age on midbrain dopaminergic and basal forebrain cholinergic neurons. First, the rat strains and husbandry conditions evaluated in prior work have varied considerably. This heterogeneity makes it difficult to generalize across studies and motivates a strain-specific characterization of dopaminergic and cholinergic neuron numbers in FBN rats. FBN rats are one of three aged rat strains supported by the United States National Institutes of Health, which renders them a valuable model for a large proportion of preclinical aging research. Second, all prior publications have compared neuron numbers in young and aged rats in only one sex. Given the sex differences in the prevalence of age-related neurodegenerative diseases that affect dopaminergic and cholinergic neurons (Cerri et al., 2019; Zhu et al., 2021), it is important to evaluate both males and females using the same approaches. To address these needs, we applied stereological sampling principles to estimate the numbers of SNc and VTA dopaminergic neurons, and medial septum and vertical limb of the diagonal band of Broca (MS/VLDB) cholinergic neurons in the brains of young adult and aged FBN rats of both sexes. The inclusion of both sexes is also supported by evidence that female rats are not more variable than male rats in neuroscience studies (Becker et al., 2016). Systematic random sampling can reduce counting bias in the X and Y planes, and z-stack acquisition can reduce overestimation due to variations in section thickness in the Z plane (West, 2012). As described below, however, the present implementation deviated from strict design-based stereology because cell profiles in image stacks were counted rather than unique cell tops using optical-disector guard zones; we therefore describe the resulting values as stereological sampling-based estimates.

## Methods

### Subjects

Fischer 344 x Brown Norway F1 hybrid rats (young female *n* = 5, aged female *n* = 6, young male *n* = 5, aged male *n* = 7) obtained from the National Institute on Aging Aged Rodent Colonies maintained by Charles River, were individually housed and kept on a 12 h reversed light/dark cycle (lights off at 0700) with access to water and food *ad libitum*. The young rats were between 5 and 6.5 months old and the aged rats were between 20 and 26 months old. All animal procedures were conducted in accordance with the University of Florida Institutional Animal Care and Use Committee and followed the guidelines of the National Institutes of Health.

### Tissue collection and processing

Animals were euthanized with 1 mL terminal sodium pentobarbital anesthesia (Euthasol, Virbac), and underwent a transcardial perfusion with cold phosphate-buffered saline (PBS, 0.01 M, pH 7.4) for 2 minutes followed by cold 4% paraformaldehyde (PFA) for 10 minutes. Brains were extracted and placed into 4% paraformaldehyde for 24 h at 4 °C. After 24 h, brains were placed in phosphate-buffered saline (PBS) at 4 °C until slicing. Serial 50-μm coronal sections were collected through the brain regions of interest using a vibratome (Vibratome Series 1000, 054018).

### Immunofluorescence

Free-floating immunofluorescence for tyrosine hydroxylase (TH) was completed as follows. Two wells of the free-floating sections (1,6) were washed in Tris-buffered saline (TBS) with 0.5% Triton X-100 (Millipore Sigma, TX1568-1) (TBS-Tx) on a shaker at room temperature. After four 5-min washes, the sections were blocked in 1% normal goat serum (NGS, Jackson ImmunoResearch Laboratories, 005-000-121) in TBS-Tx for 1 h, then incubated in primary antibody with 1% NGS and 0.5% Triton X-100 at 4 °C overnight. The primary antibody was mouse anti-TH (Millipore, AB318) at a 1:4,000 dilution. After overnight incubation, sections were washed four times for 5 min with TBS-Tx then incubated in the dark for 2 hours at room temperature with Alexa Fluor 488 goat anti-mouse secondary antibody (Invitrogen, A21121). Sections were then washed four times for 5 min with TBS-Tx in the dark. Sections were mounted with Prolong Gold Antifade Mountant (ThermoFisher, P36930), coverslipped, and allowed to dry overnight before image acquisition. Samples were stored in the dark when not being imaged.

Free-floating immunofluorescence for choline acetyltransferase (ChAT) was completed as follows. Three entire wells (1:4) of the free-floating sections were blocked in 5% normal donkey serum (NDS, Jackson ImmunoResearch Laboratories, 017-000-121) and 0.1% Triton X-100 for 2 hours at room temperature on shaker. The sections were incubated in primary antibody with 5% NDS in PBS at room temperature overnight on a shaker. The primary antibody was goat anti-ChAT (Sigma-Aldrich, AB144P) at a 1:100 dilution. The next morning, the sections were washed three times for 5 min with PBS. They were then incubated in the dark for 2 hours at room temperature with Alexa Fluor 594 donkey anti-goat secondary antibody (Invitrogen, A-11058). Sections were washed three times for 5 min with PBS in the dark. Sections were mounted with Prolong Gold Antifade Mountant, coverslipped, and allowed to dry overnight before image acquisition. Samples were stored in the dark when not being imaged.

### Region delineation

**Figure 1:**
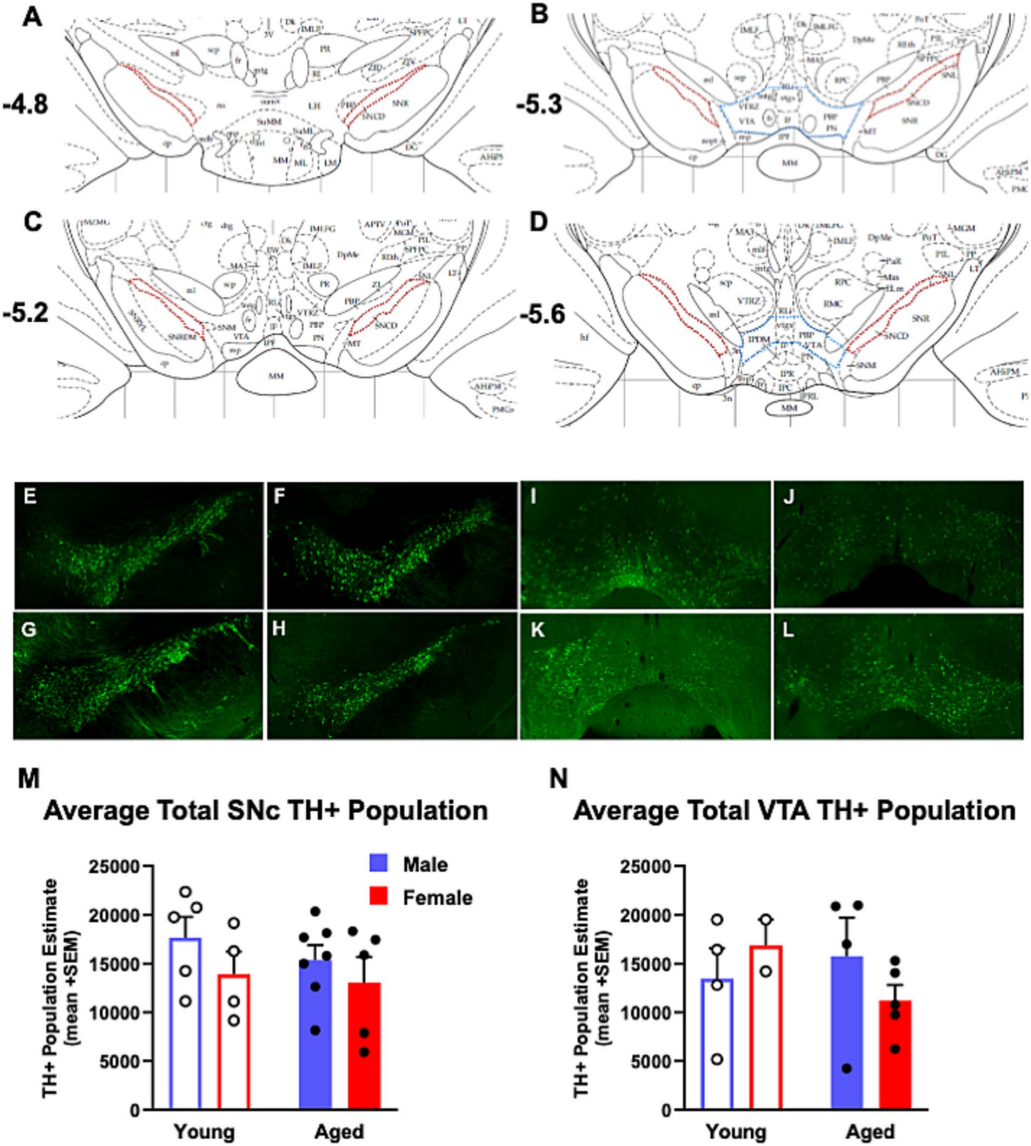
**(A–D)** Substantia nigra and ventral tegmental area boundaries used for sampling-based cell-number estimates. Illustrations from Paxinos and Watson (4th edition) showing the demarcations of midbrain dopaminergic nuclei (red outline for SNc, blue outline for VTA) used in the current study. Boundaries extend from −4.8 to −5.6 mm. The SNc is positioned dorsal to the substantia nigra pars reticulata (SNr), forming a band of densely packed neurons that extend rostrocaudally along the midbrain. In progressively caudal sections, the SNc and VTA remain contiguous, although the SNr expands ventrally and laterally. Because of the lack of clear boundaries differentiating medial SNc from the lateral VTA in these planes, fluorescent neurons within the transitional zone between SNc and VTA were included in the population estimates of each region. **(E–H)** Representative images of right SNc in a young female **(E)**, young male **(F)**, aged female **(G)**, and aged male **(H)** rat. **(I–L)** Representative images of ventral tegmental area (VTA) in a young female **(I)**, young male **(J)**, aged female **(K)**, and aged male **(L)** rat. **(M)** Average TH+ cell-number estimates for each age and sex within the SNc. **(N)** Average TH+ cell-number estimates for each age and sex within the VTA. Updated analyses should use only rat/ROI datasets with at least three usable sections and no unreplaced unusable scheduled sections. These atlas-based outlines correspond directly to the SNc and VTA contours used for stereological sampling and region delineation described in the Methods section (Region delineation), and the same criteria were applied when drawing contours on the *z*-stack images shown in panels **(E–L)**.

**Figure 2:**
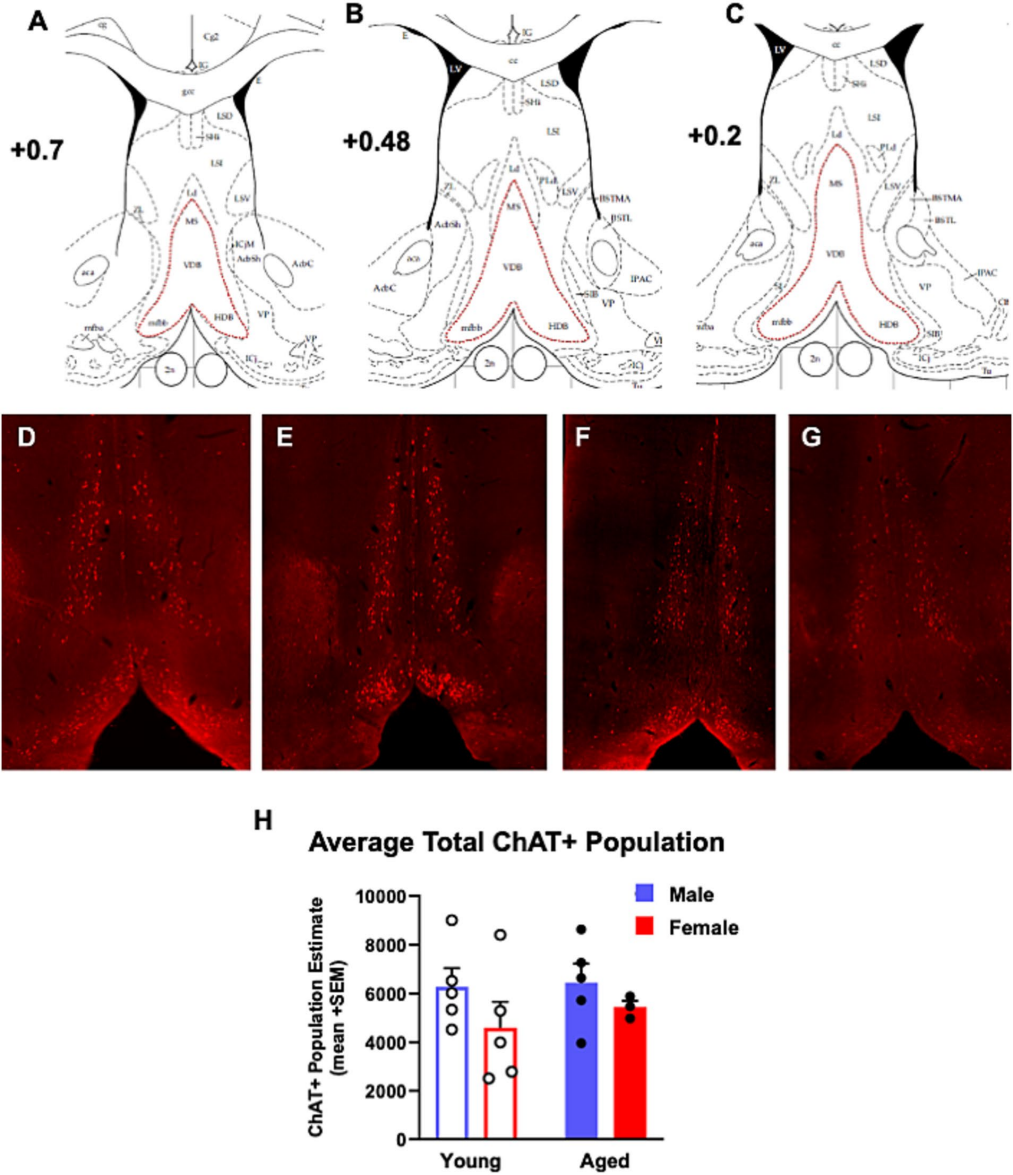
**(A–C)** Medial septum and the vertical limb of the diagonal band of Broca boundaries used for sampling-based cell-number estimates. Illustrations from Paxinos and Watson (4th edition) showing the demarcations of rostral basal forebrain cholinergic nuclei (red outline) used in the current study. Boundaries extend from +0.7 to +0.2 mm. Ventral to the MS, the VDB appears as a contiguous extension, forming a vertical column of neurons that extends toward the ventral surface of the brain. Because of the lack of clear boundaries differentiating the edge of the horizontal limb of the diagonal band of Broca, fluorescent neurons within the transitional zone were included in the population estimates of VLDB. **(D–G)** Representative images of MS and VLDB in a young female **(D)**, young male **(E)**, aged female **(F)**, and aged male **(G)** rat. **(H)** Average ChAT+ cell-number estimate for each age and sex within MS and VLDB. Updated analyses should use only rat/ROI datasets with at least three usable sections and no unreplaced unusable scheduled sections; after excluding the two aged females with insufficient MS/VLDB coverage and the aged-female outlier, the aged-female ChAT+ group should show three data points. These atlas-based outlines define the MS and VLDB contours used for stereological sampling (see Methods, Region delineation), and the same anatomical criteria were applied to the *z*-stack images shown in panels **(D–G)**.

These delineation criteria were chosen to be consistent with the atlas-based boundaries shown in Figures 1, 2 and with prior anatomical and stereological sampling studies of midbrain dopaminergic and basal forebrain cholinergic nuclei in rats that combine Paxinos and Watson coordinates with immunohistochemical markers and fiber tract landmarks.

For the medial septum/vertical limb of the diagonal band of Broca (MS/VLDB), ROIs were traced on coronal sections corresponding to +0.7 to +0.2 mm from bregma. The MS was defined as the ChAT+ cell group located immediately dorsal to the midline of the anterior commissure. The VLDB was defined as the ventral continuation of this ChAT+ cell group forming a vertical column of neurons extending toward the ventral surface of the brain along the medial wall of the hemispheres. The dorsal border of the MS was the ventral limit of the lateral septum, the ventral border of the VLDB was the point at which ChAT+ neurons merged with more diffuse labeling in the horizontal limb of the diagonal band, and the lateral borders were drawn at the lateral limits of the compact ChAT+ cell groups. Because there is no sharp cytoarchitectonic boundary between the VLDB and the horizontal limb of the diagonal band, ChAT+ neurons in this narrow transitional zone along the ventrolateral edge were included in the VLDB ROI in all animals, consistent with the demarcations illustrated in Figure 2.

Regions of interest (ROIs) for stereological sampling-based analysis were defined using a combination of the Paxinos and Watson rat brain atlas (4th edition) and the distribution of TH+ or ChAT+ immunofluorescence within each section. For the substantia nigra pars compacta (SNc) and ventral tegmental area (VTA), ROIs were traced on coronal sections corresponding to −4.8 to −5.6 mm from bregma. SNc was defined as the dense band of TH+ neurons dorsal to the TH-poor substantia nigra pars reticulata (SNr), extending rostrocaudally along the ventrolateral midbrain. The dorsal border of SNc was the transition to more sparsely labeled midbrain tissue below the red nucleus, the ventral border was the dorsal extent of SNr, and the lateral border was drawn where the dense TH+ band disappeared laterally. VTA was defined as the TH+ cell groups in the ventromedial midbrain located medial to the SNc, ventral to the red nucleus and dorsal to the interpeduncular nucleus and adjacent white matter. The medial border of the VTA was the midline, and the lateral border was drawn at the medial edge of the dense SNc band as shown in the atlas plates. In the narrow transitional zone in which TH+ neurons of SNc and VTA interdigitated and cytoarchitectonic boundaries were not clear, neurons were included in the population estimates for both regions, as described in the Figure 1 legend; the same criteria were applied in all animals.

### Image acquisition

#### Apparatus

A Zeiss Axio Imager 2 with the Apotome.2 attachment was used with a X-cite Xylis LED Light Source and a Photometrics Prime BSI USB Scientific CMOS (sCMOS) camera. Microbrightfield Bioscience Stereo Investigator software was used to establish the parameters for image acquisition and to acquire image stacks.

#### Cell counting

After completion of immunohistochemical labeling of TH and ChAT, dopaminergic and cholinergic cell bodies were estimated using a combination of StereoInvestigator-based systematic random sampling and ImageJ-based counting of acquired z-stacks. Regions of interest were mapped using the freehand contour mode within StereoInvestigator prior to placing counting frames. Contours were drawn based on a combination of the Paxinos and Watson rat brain atlas and regions of TH+/ChAT+ immunofluorescence. Parameters for image acquisition were determined by distributing a series of counting frames (100 μm × 100 μm) within a systematic random sampling (SRS) grid (300 μm × 300 μm) over the SNc, VTA, and MS/VLDB. Pilot live counts using the Optical Fractionator probe and the Resample/Oversample probe in StereoInvestigator were used to identify image-acquisition settings that were efficient while targeting a Gundersen coefficient of error < 0.1.

The final image-stack acquisition used a 1:6 section sampling interval for TH labeling and a 1:4 interval for ChAT labeling. In response to reviewer concerns about sparse sampling, revised inclusion criteria required at least three usable sections for each rat/ROI dataset. Rat/ROI datasets with fewer than three usable sections were excluded, and no two-section rat/ROI cases were retained in the revised analyses. A scheduled section was included only when the ROI could be fully delineated and imaged. If a scheduled section was unusable because of tissue damage, inadequate labeling, or imaging failure, an anatomically matched replacement section from a parallel series was used when available; if a replacement section was not available, that rat/ROI dataset was excluded rather than selectively dropping the section and altering the effective section sampling fraction.

The parameters used in the SRS Image Stack Series acquisitions were 320 μm × 326 μm for the counting frame to encompass the entire screen and 716 μm × 729 μm for the SRS grid. These parameters were used to determine the area sampling fraction. Image stacks (tiff files) were acquired with a 40 × oil immersion lens. Image stacks for each sampling site encompassed the full thickness of the section, and TH+ or ChAT+ somata were followed through the z-axis during counting. After acquisition, the tiff image files were converted to gif files, and cell counts were conducted on these images using ImageJ. The ImageJ multipoint tool was used to select distinct labeled cell bodies. Cells on the border of the image stack were counted if they crossed the top or right edge of the border and were eliminated if they crossed the left or bottom edge of the border, reflecting the same counting-frame rules used during live counting in the Optical Fractionator probe.

Because these final counts were made from labeled cell profiles visible within full-thickness image stacks, rather than by applying a strict optical-disector rule based on counting unique cell tops with guard zones, we refer to the resulting values as stereological sampling-based population estimates. At least two people (from a total of three trained Counters, all blinded to the rats’ age and sex) counted each brain’s images for both TH+ and ChAT+ labeling. For each region and animal, the total number of neurons was then estimated from the sampled counts using the scaling formula described below (Olesen et al., 2017). Representative images of the immunofluorescent staining were acquired at 20 × using the slide scanning workflow within StereoInvestigator.

#### Data analysis

For each brain region (SNc, VTA, MS/VLDB), the number of cells counted was summed between left and right hemispheres and averaged across Counters. Cell-number estimates for each brain region were compared among the three Counters to obtain metrics of inter-rater reliability using Pearson correlation coefficients. Correlations between cell counts among the three Counters had r values that ranged from 0.94 to 0.95, indicating a high degree of precision across Counters.

Total neuron number in each ROI was estimated by scaling the counted cell profiles according to the Optical Fractionator formula, *N* = ΣQ− × 1/ssf × 1/asf × 1/tsf (Olesen et al., 2017), where ΣQ− is the sum of counted neurons, ssf is the section sampling fraction (1/6 for TH+ and 1/4 for ChAT+ neurons), asf is the area sampling fraction determined by the ratio of counting-frame area (320 × 326 μm) to sampling-grid area (716 × 729 μm), and tsf is the thickness sampling fraction. In our implementation, z-stacks spanned the full thickness of each mounted section; mounted section thickness was measured at each sampling site during acquisition, and thus dissector height equaled measured section thickness (tsf = 1). We did not apply guard zones (full-thickness sampling); all sites were processed identically across groups, and inclusion/exclusion rules were applied consistently across the z-axis.

To quantify sampling precision, Gundersen coefficients of error (CE; *m* = 1) were computed for each included animal and region using StereoInvestigator, and coefficients of variation (CV = SD/mean) were computed across animals within each group; section counts, final inclusion/exclusion status, and summary CE/CV values for SNc, VTA, and MS/VLDB are provided in Supplementary Table 1 (Gundersen et al., 1999; West, 1999). Because the Gundersen CE model is most reliable when the sampled ROI is represented by a greater number of sections, and because the reliability of section-to-section variance estimates is limited with only three to five sections, CE values from the present sparse sampling design should be interpreted cautiously. CE values were not calculated for excluded two-section rat/ROI cases.

### Statistical analyses

Statistical analyses were conducted using GraphPad Prism 10 (GraphPad Software, San Diego, CA, USA). Outliers within each age and sex group were determined by the interquartile range method, such that for each brain region, data points that fell below or above 1.5 times the interquartile range of all the data were considered outliers and excluded. Analyses were conducted after applying the revised rat/ROI inclusion criterion requiring at least three usable sections and after excluding rat/ROI datasets with unreplaced unusable scheduled sections. Using the outlier criterion, TH + and ChAT+ cell counts from one rat (aged female) fell above 1.5 times the interquartile range and were excluded from further analysis. Cell-number estimates in each brain region were then compared between groups using two-factor ANOVA, with Age and Sex as between-subjects variables. Additional analyses employed Pearson correlations to evaluate relationships between cell-number estimates in different brain regions, as well as between cell-number estimates and exact ages. Statistical significance was set at *p* < 0.05 for all analyses.

## Results

### Dopaminergic cells

Figure 1 shows data from young and aged rats’ TH+ stereological sampling-based cell-number estimates. Figures 1A–D show the borders of the SNc and VTA used for estimation, and Figures 1E–H,I–L show representative images of TH+ cells in SNc and VTA, respectively, for rats in each age and sex condition. Final sample sizes for TH+ analyses in SNc were: young female *n* = 4, young male *n* = 5, aged female *n* = 5, aged male *n* = 7, and in VTA were: young female *n* = 2, young male *n* = 4, aged female *n* = 5, aged male *n* = 4. Evaluation of the effects of Age and Sex on estimated TH+ cell numbers in the SNc via two-factor ANOVA revealed no main effects of Age [*F*(1,17) = 0.54, *p* = 0.47] or Sex [F(1,17) = 2.03, *p* = 0.17], nor was there an interaction between the two variables [F(1,17) = 0.11, *p* = 0.74; Figure 1M]. A similar analysis of estimated TH+ cell numbers in the VTA also revealed no main effects of Age [*F*(1,11) = 0.28, *p* = 0.61] or Sex [F(1,11) = 0.03, *p* = 0.86], nor was there an interaction between the two variables [F(1,11) = 1.56, *p* = 0.24; Figure 1N]. Further comparison of cell counts between SNc and VTA revealed a significant correlation in counts between the two regions [r(15) = 0.56, *p* = 0.03], but no relationships between cell numbers and age in months for either brain region [SNc: r(21) = −0.10, *p* = 0.67; VTA: r(15) = −0.10, *p* = 0.73]. Considered together, these data suggest that dopaminergic midbrain neuron numbers in the FBN rat SNc and VTA are spared in aging, and that such sparing is equivalent in both sexes and comparable across both cell groups.

### Cholinergic cells

Figure 2 shows data from young and aged rats’ ChAT+ stereological sampling-based cell-number estimates. Figures 2A–C show the borders of the MS/VLDB used for estimation, and Figures 2D–G show representative images of ChAT+ cells in this region for rats in each age and sex condition. Final sample sizes for ChAT+ analyses were: young female *n* = 5, young male *n* = 5, aged female *n* = 3, aged male *n* = 5. Evaluation of the effects of Age and Sex on estimated ChAT+ cell numbers in the MS/VLDB via two-factor ANOVA revealed no main effects of Age (*F*(1,14) = 0.33, *p* = 0.58) or Sex (F(1,14) = 2.30, *p* = 0.15), nor was there an interaction between the two variables (F(1,14) = 0.15, *p* = 0.70; Figure 2H). Further comparisons of MS/VLDB cell counts with those in SNc and VTA revealed no correlations between cholinergic and dopaminergic cell numbers (SNc: r(16) = 0.36, *p* = 0.17; VTA: r(12) = −0.15, *p* = 0.65), nor was there a relationship between MS/VLDB cell numbers and age in months (r(18) = 0.24, *p* = 0.35). These data suggest that cholinergic neuron numbers in the FBN rat MS/VLDB are spared in aging, that such sparing is equivalent in both sexes, and that basal forebrain cholinergic cell numbers are orthogonal to midbrain dopaminergic cell numbers.

## Discussion

The goal of this study was to estimate dopaminergic neurons in the SNc and VTA, and cholinergic neurons in the MS/VLDB in young adult and aged FBN rats of both sexes, as previous quantification of these cell types in aged rats has varied and focused on only one sex. The results indicate no large differences in estimates of either cell type as a function of age or sex in either brain region, although the revised analyses should be interpreted in light of the sparse section sampling and the fact that the final workflow used stereological sampling principles rather than strict design-based stereological quantification. Such data will be valuable for future research in this rat strain because they can serve as a baseline against which models of age-related neurodegenerative diseases such as PD and AD can be compared (e.g., Jagmag et al., 2016).

Given the modest sample sizes in each age-by-sex group after revised exclusions, it is also important to consider the sensitivity of the present design. Based on the observed variability in neuron counts and standard power considerations for a 2 × 2 between-subjects ANOVA with *α* = 0.05, the study is expected to have adequate power to detect medium-to-large main effects of age or sex on neuron-number estimates, but reduced power to detect smaller effects. Thus, the absence of statistically significant group differences should be interpreted as evidence against large age- or sex-dependent changes in SNc, VTA, or MS/VLDB neuron-number estimates, while more subtle effects cannot be ruled out. In addition, the current sectioning fractions resulted in sparse ROI sampling, typically only three to five sections per included rat/ROI after revised exclusions. Increasing the sampling frequency, for example by changing the TH section sampling interval from 1:6 to 1:3 and the ChAT interval from 1:4 to 1:2, would increase the number of sections to approximately five to eight per ROI in many animals and would likely improve both statistical power and CE reliability.

An additional limitation is that our analyses focused on neuron-number estimates. We did not quantify somatic size, dendritic morphology, marker intensity, axonal/terminal density, or synaptic markers, which can show age-related alterations even when total neuron number is preserved (e.g., Emerich et al., 1993; Pereira et al., 2021). Future work in FBN rats should pair more intensive design-based stereology with morphometric and connectivity measures to determine whether more subtle structural changes occur in these systems across aging.

In previous studies quantifying dopaminergic and cholinergic cell numbers in young and aged rats, there has been disagreement about whether cell loss occurs with age and, if so, the timepoint at which such loss occurs. The present study contributes to this literature by adding information about the lack of large differences observed between 5–6.5-month-old and 20–26-month-old FBN rats. One explanation for discrepancies in the literature is variability in rat strain, age range, ROI definition, staining/thresholding criteria, and sampling methodology. While some prior dopaminergic cell counts used Sprague–Dawley rats (Emerich et al., 1993; Gao et al., 2011; Sanchez et al., 2008), others used Fischer 344 (Allard et al., 2011), Wistar (Pereira et al., 2021), and FBN (Mizoguchi et al., 2009) strains. Within the cholinergic neuronal population literature, studies have used Sprague Dawley (de Lacalle et al., 1996), Wistar (Niewiadomska et al., 2000; Pereira et al., 2021), Fischer 344 (Bañuelos et al., 2013), FBN (Casu, 2002), and Long-Evans (Baskerville et al., 2006) strains. In addition, several approaches, including non-stereological methods and stereological sampling workflows of varying intensity, have been used to quantify cell numbers, making it difficult to reach a unified conclusion regarding the impact of age on cell numbers.

The comparison with Bañuelos et al. (2013) is particularly important because that study reported approximately three-fold more ChAT+ neurons in the MS/VLDB region than the present estimates. Although methodological differences may contribute to this discrepancy, the approaches were not entirely dissimilar; both studies used ChAT immunolabeling and sampling-based cell counts in rostral basal forebrain regions. Important differences include rat strain (Fischer 344 in Bañuelos et al. (2013) versus FBN in the current study), age and behavioral cohort characteristics, exact anatomical boundaries, immunohistochemical and imaging details, and sampling intensity. The higher sampling intensity in Bañuelos et al. (2013) may have yielded more stable absolute estimates, whereas the sparse sampling used here may reduce precision and increase sensitivity to section-level variability. Thus, the difference in absolute counts may reflect a combination of methodological factors and true biological differences between strains or cohorts. For this reason, the present data are most appropriately interpreted as within-study comparisons of age and sex under a common sampling workflow, rather than as definitive absolute totals to be compared directly between studies.

One possible explanation for the lack of age differences in dopaminergic and cholinergic cell populations in the current study is that vulnerability of these systems may vary across individuals, such that some aged FBN rats retain relatively preserved neuron-number estimates despite functional decline, paralleling the individual variability in nigral and basal forebrain pathology described in humans (e.g., Buchman et al., 2012; Grothe et al., 2013). Resilience refers to the ability to display intact function in the presence of adverse effects such as age, stress, or pathology. In FBN rats in particular, cognitive performance exists on a spectrum, with some rats performing well at advanced ages and others exhibiting impairments. For example, in a study by McQuail and Nicolle (2015) in which FBN rats of various ages were evaluated for spatial memory, 18-month rats as a group were impaired relative to 6-month rats, and 24-month rats were impaired relative to both 6- and 18-month rats. Nevertheless, roughly 50% of the 24-month rats still performed similarly to 6-month rats. Similar results are evident in studies of aged Long Evans and Fischer 344 rats in spatial memory (LaSarge et al., 2007; Gallagher et al., 1993) and executive functioning tasks (Beas et al., 2013) and olfactory discrimination/perceptual similarity learning (Yoder et al., 2017). In the current study, the cell-number estimates of dopaminergic and cholinergic cells varied between rats, suggesting that some rats may be more susceptible to cell loss with age while others are more resistant, although the fact that variability in cell-number estimates was comparable in both age groups argues against this interpretation. It will be important in future studies to determine the extent to which variability in cell number at all ages is related to cognitive performance, such as in previous studies that evaluated associations between cholinergic neuron numbers and spatial learning abilities (Baskerville et al., 2006; Bañuelos et al., 2013).

Another possible explanation for the lack of age-related declines in cell numbers is that the aged rats in this study were simply not old enough. In a prior study (Sanchez et al., 2008), the number of TH+ neurons in the SNc and VTA were evaluated in young (4–6 months), old (22–24 months), and very old (30–32 months) female Sprague Dawley rats. No differences were observed when comparing young and old rats, but there was a significant reduction in SNc cell numbers in very old compared to young rats. This decrease in the oldest age group suggests that if older rats were included in the current study, a reduction in (at least SNc) neuron number may have been observed. One limitation with this approach, however, is the survival of the FBN rats as they age. While 90% of FBN males and females survive at 23 months and 25 months respectively, only 75% of FBN males and 50% of FBN females survive at 30 months (Turturro et al., 1999). This may create a need for a larger number of rats in the study initially and more careful monitoring of the very old rats, as well as consideration of the possibility of survivor bias. Notably, cognitive deficits at the group level are present in aged compared to young FBN rats at the ages evaluated in the current study (McQuail and Nicolle, 2015; Hernandez et al., 2017; Johnson et al., 2017; Smith et al., 2022), indicating that these ages are sufficiently old to model cognitive impairments observed in aged humans in the absence of neurodegenerative disease.

### Sex differences in dopaminergic and cholinergic cells

In addition to the absence of age differences, there were also no clear sex differences in dopaminergic and cholinergic cell-number estimates. Several prior studies have quantified dopaminergic and cholinergic markers in male and female rats and found varying degrees of sex differences. For example, Miller (1983) found that cycling female rats have fewer dopamine receptors than males. Duchesne et al. (2009) used high performance liquid chromatography to show that female rats have more dopamine in ventral medial prefrontal cortex whereas males have more dopamine metabolites, and Abdel-Hafez and Mohamed (2013) found that, compared to males, aged female albino rats have more dopaminergic neurons in the substantia nigra. Regarding cholinergic neurons, Luine et al. (1986) found that aged female rats have 30% less ChAT activity and 40% less AChE activity compared to their age-matched male counterparts, while Veng et al. (2003) found that these neurons in young male rats have a larger surface area compared to age-matched females. The current study suggests that despite the presence of sex differences in some markers of dopaminergic and cholinergic function, the numbers of these neurons (at least in FBN rats) do not show a large sex-dependent difference under the present sampling workflow. It will be important in future research to continue to include both males and females given the strong sex disparities in PD and AD, in which these neuron populations are vulnerable (Cerri et al., 2019; Zhu et al., 2021). In addition, it is important to consider that unlike humans and some strains of rats (e.g., Sprague–Dawley), female FBN rats maintain estrous cyclicity at advanced ages (LeFevre and McClintock, 1988; Hernandez et al., 2020). As such, it will be important to consider whether and how gonadal hormone status may influence cell numbers at advanced ages.

## Conclusion

Male and female Fischer 344 x Brown Norway F1 hybrid rats at young (5–6.5 months) and aged (20–26 months) timepoints were evaluated for stereological sampling-based estimates of dopaminergic cells in the SNc and VTA, and cholinergic cells in the MS/VLDB. Within the limitations of sparse ROI sampling and a cell-profile counting workflow, the results showed no large differences as a function of age or sex in estimates of either cell type, although future work should use higher sampling intensity, consider even older ages, and collect concurrent functional/behavioral measures that can potentially be linked to cell-count data.

## Data Availability

The raw data supporting the conclusions of this article will be made available by the authors, without undue reservation.

## Supplementary material

SUPPLEMENTARY TABLE 1 Section counts, final inclusion/exclusion status, Gundersen coefficient of error (CE; m = 1) for each included animal and region, and coefficient of variation (CV) across animals within each group. The Gundersen coefficient of error was calculated using the Stereo Investigator implementation of the Gundersen CE model, with Qi representing the count in section i, **A** representing the sum of squared counts, **B** representing the covariance of adjacent sections, and **C** representing the covariance of sections two apart. Coefficient of variation was calculated by dividing the group standard deviation by the group mean. Rat/ROI datasets with fewer than three usable sections, or with a scheduled section that was unusable and could not be replaced with an anatomically matched section from a parallel series, were excluded from the revised analyses; CE values were not calculated for excluded two-section cases.
