# Supplementary material for: Stereological sampling-based estimates of dopaminergic and cholinergic neuron numbers in young adult and aged male and female Fischer 344 × Brown Norway F1 hybrid rats
Source: Front Aging Neurosci. 2026 Aug 3;18:1730515. doi: 10.3389/fnagi.2026.1730515 (PMC13478069; doi:10.3389/fnagi.2026.1730515)
Supplement: Supplementary file 1 [file Data_Sheet_1.pdf]

Rat ID	sex	age	SNc CE	# sections	VTA CE	# sections	MS/VLDB CE	# sections	SNc CV	VTA CE	MS/VLDB CV
HEP4	m	y	0.071	3	x	x	0.072	5	0.269	0.458	0.270
HEP7	m	y	0.051	5	0.053	4	0.052	4			
HEP15	m	y	0.053	4	0.055	4	0.062	3			
HEP16	m	y	0.048	4	0.051	4	0.067	4			
HEP18	m	y	0.081	3	0.095	3	0.063	3			
HEP2	f	y	0.060	3	x	x	0.094	4			
HEP8	f	y	0.055	4	0.052	3	0.048	4	0.330	0.222	0.522
HEP17	f	y	0.078	3	0.059	3	0.075	3			
HEP23	f	y	0.070	3	x	x	0.087	4			
HEP27	f	y	x	x	x	x	0.067	4			
HEP3	m	o	0.072	4	0.047	4	0.083	3	0.261	0.502	0.271
HEP6	m	o	0.047	5	0.052	5	0.057	4			
HEP14	m	o	0.078	3	x	x	x	x			
HEP19	m	o	0.055	4	0.051	4	0.064	3			
HEP20	m	o	0.064	3	x	x	0.070	3			
HEP21	m	o	0.049	5	0.074	4	0.068	4			
HEP24	m	o	0.076	3	x	x	x	x			
HEP5	f	o	0.046	3	0.048	3	x	x	0.439	0.350	0.083
HEP25	f	o	0.060	4	0.056	3	x	x			
HEP26	f	o	0.025	4	0.060	3	x	x			
HEP28	f	o	0.075	3	0.091	3	0.072	4			
HEP29	f	o	x	x	x	x	0.071	4			
HEP30	f	o	0.088	3	0.071	3	0.055	4			
